# Heat transfer model for deep tissue injury: a step towards an early thermographic diagnostic capability

**DOI:** 10.1186/1746-1596-9-36

**Published:** 2014-02-20

**Authors:** Akanksha Bhargava, Arjun Chanmugam, Cila Herman

**Affiliations:** 1 Department of Mechanical Engineering, The Johns Hopkins University, Baltimore, MD, USA; 2 Department of Emergency Medicine, The Johns Hopkins University School of Medicine, Baltimore, MD, USA

**Keywords:** Deep tissue injury (DTI), Ischemia, Inflammation, Heat transfer model, Computational model, Skin surface temperature, Early diagnosis, Infrared (IR) thermography

## Abstract

**Background:**

Deep tissue injury (DTI) is a class of serious lesions which develop in the deep tissue layers as a result of sustained tissue loading or pressure-induced ischemic injury. DTI lesions often do not become visible on the skin surface until the injury reaches an advanced stage, making their early detection a challenging task.

**Theory:**

Early diagnosis leading to early treatment mitigates the progression of the lesion and remains one of the priorities in clinical care. The aim of the study is to relate changes in tissue temperature with key physiological changes occurring at the tissue level to develop criteria for the detection of incipient DTIs.

**Method:**

Skin surface temperature distributions of the damaged tissue were analyzed using a multilayer tissue model. Thermal response of the skin surface to a cooling stress, was computed for deep tissue inflammation and deep tissue ischemia, and then compared with computed skin temperature of healthy tissue.

**Results:**

For a deep lesion situated in muscle and fat layers, measurable skin temperature differences were observed within the first five minutes of thermal recovery period including temperature increases between 0.25°C to 0.9°C during inflammation and temperature decreases between −0.2°C to −0.5°C during ischemia.

**Conclusions:**

The computational thermal models can explain previously published thermographic findings related to DTIs and pressure ulcers. It is concluded that infrared thermography can be used as an objective, non-invasive and quantitative means of early DTI diagnosis.

**Virtual slides:**

The virtual slides for this article can be found here: http://www.diagnosticpathology.diagnomx.eu/vs/1461254346108378.

## Introduction

Deep Tissue Injury (DTI) is a type of pressure injury that first arises in the deeper tissue with the overlying skin remaining intact. DTI lesions can progress rapidly and can be difficult to manage if not recognized early, causing increased morbidity and mortality among pressure ulcer patients. The costs to individuals and to society of pressure injuries increased substantially, from $2.2 to $3.6 billion per year in 1999 to more than $11 billion in 2011 [1,2]. The difficulty with early detection of DTI lesions is that they are poorly visible until substantial soft tissue damage has occurred.

Although DTI has been referred to in the medical literature since the 1800s [3], it has received relatively little attention in the medical community. Shea, who was credited for labeling the four stage system of pressure ulcers on the basis of the extent of soft tissue damage in 1975, described DTIs as innocent appearing lesions that are potentially fatal [3]. However, it was only in 2007 when this injury pattern was formally recognized by The National Pressure Ulcer Advisory Panel (NPUAP) [4]. The NPUAP’s updated pressure ulcer classification system relies on visible characteristics of skin damage. It includes stage I through IV pressure ulcers (with stage I ulcers defined as the least severe - to stage IV ulcers, which involve full tissue thickness loss), unstageable pressure ulcers and DTIs [4]. A DTI lesion, by definition, is identified as a skin lesion with intact skin, which is likely to evolve into an advanced stage III or stage IV pressure ulcer [5]. Visible characteristics of the injury may not appear on the skin surface until there is substantial damage to the underlying tissue [5]. The mechanism behind the progression of a DTI is likely multifactorial, and may be related to a combination of direct ischemic injury and indirect ischemia-reperfusion injury with production of cytotoxic and inflammatory mediators.

In view of above definitions, early injury detection and appropriate preventive intervention, especially when there are no visible signs of injury, are the most effective management methods for DTIs. The challenge is to diagnose damage in the deeper tissue early, when there are few visible signs of tissue damage on the skin surface. As an example, nonblanchable erythema of the intact skin is a characteristic that the NPUAP associates with stage I pressure ulcers, but it may also be a sign of a deeper tissue damage that occurs in DTIs [5]. Therefore, the proper assessment of a wound (that presents itself with minimal skin changes or even with nonblanchable erythema), can prevent misclassification of rapidly progressing DTIs as more innocent appearing stage I pressure ulcers. The focus of the research groups is to evaluate different techniques that can provide early DTI detection. Ultrasonography is considered as one of the most suitable techniques for detecting the extent of soft tissue damage in DTI [5]. However, current diagnostic techniques use a qualitative approach and involve subjective interpretation of results. Certain biomolecules have been tested as biomarkers of tissue damage in animal DTI models, for establishing greater diagnostic accuracy, but their applicability is yet to be examined on human subjects [5]. The goal of this study is to develop a computational thermal model (that explains to explain the previously published, seemingly inconsistent thermographic data) which will account for the pathophysiological changes occurring in DTI and will provide a means of early injury detection. Even more important is that this model can serve as the foundation for a more rigorous, quantitative interpretation of other soft tissue thermographic images, which can lead to more exact quantitative detection and diagnostic criteria.

## Background

Although infrared (IR) thermography has been attempted for the diagnosis of pressure related injury since 1970s, the results of the studies were inconclusive. Different thermal patterns were observed in thermographic images that showed skin tissue recovery from an external pressure application [6]. In some of the thermograms, the region, that was under pressure, appeared hotter than the surrounding skin and it appeared colder in others. Measurement of skin temperatures using thermocouples also produced similar results [7]. While some studies reported an increase in skin temperature [8-10], there were others who reported temperature decrease at the site of pressure injury [11,12]. These inconsistent observations can be explained partly by the subjective interpretation of images and partly by the limited insight into underlying pathophysiological processes that cause changes in skin temperature. Because of the seemingly inconsistent data (both increase and decrease in temperature in addition to normal temperature distributions), thermography was abandoned for the diagnosis of DTI. The sequential progression of ischemia and inflammation and the associated temperature changes may not have been fully appreciated in these prior studies and this is one of the topics addressed in the present paper.

Computational modeling allows the analysis of a variety of biophysical processes relatively inexpensively, in order to improve the understanding of the underlying physical and chemical processes for a range of system parameters and properties. These models allow the investigator to evaluate the influence of specific variables on the surface temperature distributions that can be measured with IR thermography. We postulate that computational models for heat transfer in deep tissue injury can facilitate the interpretation of thermographic images and can therefore help to improve its diagnostic capability by decreasing reliance on subjective interpretation.

Our quantitative heat transfer model for deep tissue lesions explains the seemingly inconsistent temperature fluctuations which were previously reported in the literature for pressure ulcers [6-12]. Notably, this model uses ischemia and inflammation as predominant variables to explain the temperature fluctuations associated with DTIs. We believe this is the first step in creating a valid quantitative DTI model, that can be used to perform parametric and sensitivity analyses. The model also lays the foundation for upcoming clinical trials.

In this paper, we first propose two sequences of underlying biophysical and chemical processes that lead to both ischemia mediated and ischemia-reperfusion mediated damage in DTI. In both cases, we identify the processes leading to temperature changes (either increase or decrease). Next, a mathematical model describing the heat transfer processes in the tissue of the heel is introduced. The model is used to demonstrate the temperature changes in the heel tissue for a range of lesion depths, thermophysical properties and external conditions. The results explain the influence of specific parameters on the skin surface temperature distribution – which can be measured non-invasively by IR thermography. They also elucidate the role of ischemia and inflammation in the development of DTI.

## Tissue physiology in DTI

**Figure 1:**
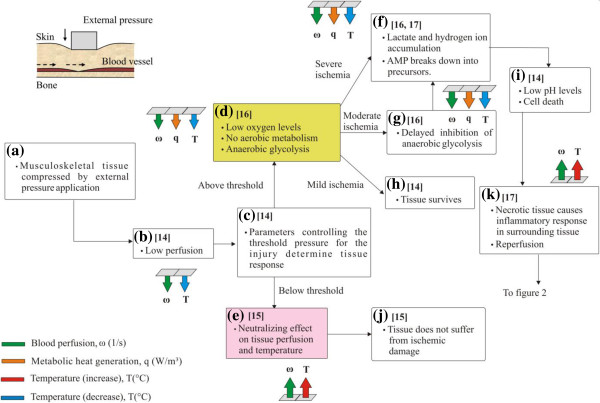
**Ischemia mediated DTI mechanism and physiological responses of the affected tissue.** The responses are related to changes in perfusion *ω* (marked green), metabolic heat generation rate *q* (marked orange) and temperature *T* (marked red to indicate increase and blue to indicate decrease). An arrow pointing up indicates an increasing trend in a parameter and vice versa. Blocks denote key events and the arrows indicate paths between the events. Relevant references are shown in each block.

**Figure 2:**
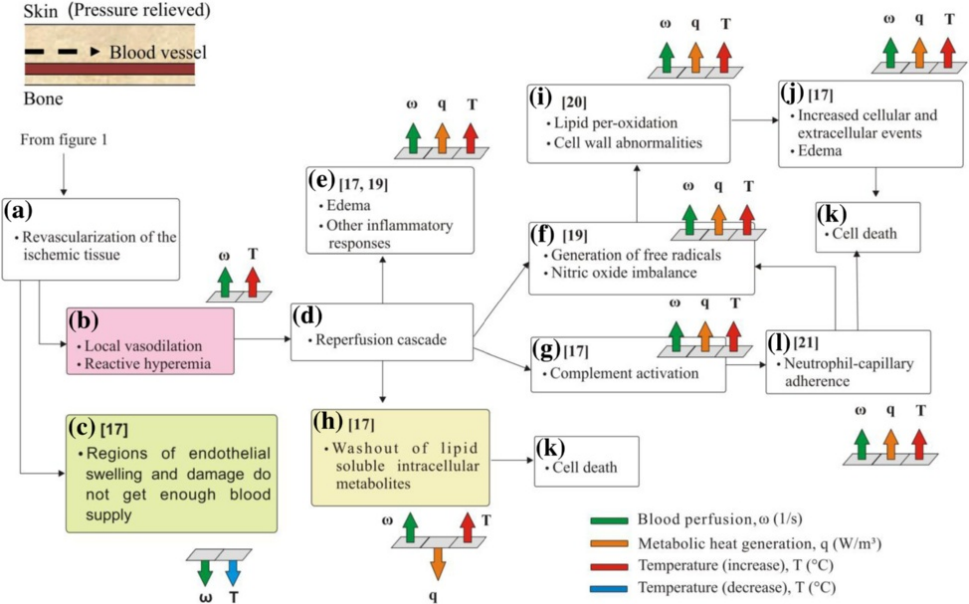
**Ischemia reperfusion mediated DTI mechanism and physiological responses of the affected tissue.** The responses are related to changes in perfusion *ω* (marked green), metabolic heat generation rate *q* (marked orange) and temperature *T* (marked red to indicate increase and blue to indicate decrease). An arrow pointing up indicates an increasing trend in a parameter and vice versa. Blocks denote key events and the arrows indicate paths between the events. Relevant references are shown in each block.

Based on findings from the literature, the sequences of events that lead to tissue damage in DTI are presented schematically in Figure 1 for ischemia and in Figure 2 for ischemia-reperfusion mediated damage. The rectangular blocks in Figures 1 and 2 indicate characteristic events and associated physiological response mechanisms. Literature sources describing a particular event are indicated in each block. The arrows connecting the blocks form pathways that connect a series of events. The injury mechanisms and physiological responses of the affected tissue introduced in Figures 1 and 2 are related to changes of blood perfusion *ω,* metabolic heat generation rate *q* and temperature *T*. The magnitude of these parameters is indicated by groups of arrows next to the blocks, with an arrow pointing up suggesting an increasing trend and vice versa. The changes of these parameters will be tied to results from our modeling efforts, as well as to thermographic findings in future research.

### Ischemia mediated damage in DTI

Application of external pressure compresses the musculoskeletal tissue and causes skin perfusion to decrease locally in the affected region (Figure 1a). This also causes an initial decrease in skin temperature (Figure 1b). The state of the tissue is described as ischemia as the tissue experiences transient partial or complete loss of blood supply [13]. Whether or not the tissue experiences any permanent damage as a result of this reduced perfusion depends on various factors (Figure 1c). The main determinants include the magnitude of compressive forces, the duration of loading, the baseline perfusion activity of the tissue and the vascular integrity, as well as the immunocompetence of the patient. All these factors play an important role in deciding the threshold for the onset of tissue damage [14]. If the pressure disturbance is below the threshold, thermoregulation processes are able to restore oxygen and other nutrients by adjusting the perfusion rate in the tissue, thereby allowing the tissue to recover its baseline state without suffering from ischemic damage [15]. Removal of a tolerable pressure (a pressure less than the damage threshold), should allow the tissue to regain its normal temperature or attain a slightly higher temperature than the surrounding tissue due to vasodilation and resultant hyperemia (especially if there are increases in baseline perfusion levels during the post injury modified thermoregulation process) (Figure 1e, Figure 1j [15]).

Pressure levels above the tolerable pressure limit (above threshold) will initiate a sequence of biochemical changes that are capable of causing tissue damage and cell necrosis. Ischemic cells lacking sufficient oxygen to continue aerobic metabolism will resort to anaerobic metabolic processes for ATP generation [16]. The rate at which the energy is generated during anaerobic metabolism is much lower than the rate at which it is consumed by the cells, resulting in an overall decrease in ATP levels and decreased heat generation inside the tissue [16,17]. Because of reduced perfusion levels and decrease in metabolic heat generation, tissue temperatures are lower than the surrounding tissue (Figure 1d).

The extent of tissue damage varies with the severity of ischemic conditions [16]. Cell necrosis is possible within 30 minutes under severe ischemic conditions (blood flow levels are less than 10 percent of the control) (Figure 1f). Under moderate ischemic conditions (blood flow levels are between 10–35 percent of the control), tissue necrosis can also occur if perfusion impairment persists for a sufficiently long duration, usually longer than the previous case (Figure 1g). The temperature decrease is expected to be more pronounced in cases of severe ischemia than moderate ischemia. The lowest temperatures of the ischemic cycle should occur in conditions of severe or moderate ischemia, especially when the anaerobic metabolism has been exhausted (Figure 1i). When subjected to mild ischemic conditions (blood flow levels are more than 35 percent of the control), tissue may withstand diminished perfusion levels and the accumulated impact of anaerobic metabolism long enough to avoid cell death (Figure 1h). In this post injury state, the recovered tissue will either attain its normal temperature or will have higher than normal temperatures.

### Ischemia-reperfusion mediated damage in DTI

Repeated ischemic insults, resulting in subsequent ischemia-reperfusion cycles, hold the potential of causing greater tissue damage than a single ischemic insult [18], as illustrated in Figure 2. During post-ischemic tissue recovery, revascularization occurs in the previously ischemic tissue (Figure 2a). Local vasodilation increases tissue blood perfusion, thereby causing reactive hyperemia and a temperature increase (Figure 2b). Endothelial injury from ischemia may cause local swelling and diminished flow and limit the reactive hyperemia in some parts of injured tissue [17]. Tissue temperature in such regions is expected to remain same as that of the previously ischemic tissue (Figure 2c).

During revascularization, oxygen re-enters the hypoxic tissue [17] (Figure 2d) which initiates several potential cell damaging pathways [19,20]. This includes generation and accumulation of reactive oxygen species (ROS), reduction in nitric oxide levels (Figure 2f), and activation of complement cascade (Figure 2g) [19,20]. Imbalance occurs in tissue superoxide-nitric oxide levels with increased accumulation of ROS species and decreased concentrations of nitric oxide levels [19] (Figure 2f). Activation of the complement cascade stimulates local endothelial-white blood cell adhesion [17,21] (Figure 2g,l), thereby releasing cytotoxic enzymes and more oxygen free radicals (Figure 2f). These changes can cause lipid per-oxidation, edema and cell wall abnormalities resulting in cell death (Figure 2i-k) [19].

In addition to the mechanisms already discussed, increased delivery of substrates can cause washout of lipid soluble intracellular metabolites (such as lactate molecules and precursors of adenine nucleotide formation) (Figure 2h) which could also result in cell death (Figure 2k) [17]. An increased volume of cellular and intracellular fluid, also a result of increased blood perfusion, results in edema and aids in further release of inflammatory mediators along with recruitment of white blood cells [17] (Figure 2e). This increased perfusion along with the release of inflammatory mediators and increased white cell activity results in an increased temperature, which can be detected thermographically. It should be noted that the accumulated impact of multiple ischemia-reperfusion cycles is likely to result in more cell injury and death when compared to a single ischemic insult.

### Heel DTI

In this work, we present a computational thermal model of heel tissue as a representative thermal model of a DTI. By obtaining thermographic measurements of an injured heel and comparing measurement data to data computed using this model, clinicians can gain better quantitative insight into the properties of the DTI.

### Heel anatomy and vascularity

Tissue perfusion is necessary to enable much needed oxygen delivery as well as the removal of the products of metabolism, and is one of the main determinants of tissue viability. Perfusion is mediated through the central nervous system which adjusts blood flow to meet tissue metabolic demands. Several factors, such as temperature, pain, external pressure, arterial and venous functions, can all affect tissue perfusion. If tissue perfusion is inadequate, cell death can occur, resulting in deep tissue injury.

**Figure 3:**
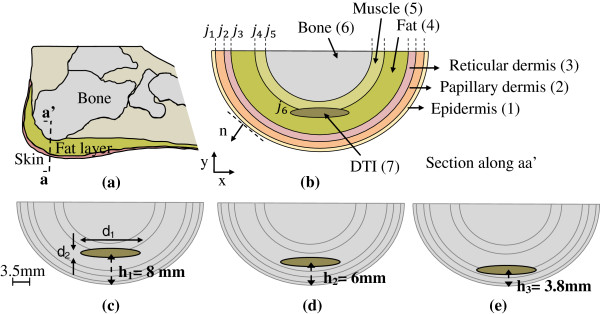
**Schematic of the heel tissue and the computational domain for three lesion depths. (a)** Cross section of the heel with aa’ as the projection of the computational domain. **(b)** Computational domain (across section aa’) with tissue layers and the DTI lesion. DTI lesion at depth **(c)** h_1_=8 mm (in the fat and muscle layer), **(d)** h_2_=6 mm (in the fat layer) and **(e)** h_3_=3.8 mm (in the fat layer and reticular dermis). Lesion dimensions: d_1_ = 1.5 cm, d_2_ = 0.25 cm.

The heel is one of the most common sites where DTIs occur. It is composed of the talus bone, a thick and large bone that comprises the posterior aspect of the foot (Figure 3a). The inferior portion of the heel is covered by thick skin and is well padded by a well-supported structure, the cup ligament. The posterior aspect of the heel, however, lacks this thick padding and also the rich vascularity that characterizes the inferior portion.

Although the heel is equipped to handle the pressure of walking, running or standing, prolonged immobility in chronically ill patients can result in external pressures of long duration to parts of the heel that normally are not subject to prolonged pressure. When an individual is recumbent for extended periods during hospitalization or a profound disability, it is the posterior aspect of the heel that is primarily subjected to prolonged compression against the talus bone. Interestingly, the posterior aspect of the heel has the least amount of soft tissue and also has the least vascularity. As the compression continues for an extended period, this low vascularized tissue develops risk for ischemia and deep tissue injury. This is especially the case among hospitalized patients where heel DTIs cause significant morbidity [22].

## Methods

Although thermographic imaging of DTI was attempted in the past as a means of early diagnosis, the results appeared inconclusive. Some studies indicated temperature increase [8-10] while others showed temperature decrease [11,12]. In our computational thermal model, we introduce ischemia and inflammation as stages of DTI to explain the previously obtained seemingly contradictory temperature findings.

To better quantify the thermal signatures associated with ischemic and reperfusion processes, our heel model included both a steady state analysis (corresponding to data for steady state infrared imaging) and a transient analysis (matches the conditions in dynamic infrared imaging). In the steady state analysis the skin surface is exposed to ambient conditions. The goal of the transient analysis is to enhance the temperature differences between healthy and diseased tissue (when compared to the steady state situation), which can yield a stronger measurement signal in a clinical application. Therefore, the understanding of the transient response of the tissue is critical. The transient analysis begins with the skin (heel) exposed to ambient conditions such as in the steady state analysis. At time *t* = 0 cooling is applied to the skin surface (constant surface temperature of 15°C for the duration of one minute in the present study). In a clinical setting, this can be accomplished by applying a gel pack at 15°C to the skin surface. The duration of cooling and the cooling temperature can be optimized for clinical applications to minimize patient discomfort while achieving maximum temperature differences. After the cooling is removed, the tissue is again exposed to ambient conditions and it gradually warms up to reach steady state temperature. This reheating process is called the thermal recovery. Our goal is to demonstrate that the thermal recovery of tissue previously subjected to cooling (which was previously used in detecting melanoma [22]) would result in a characteristic response (thermal signature) yielding a capability to quantitatively detect deep tissue injury, both at the ischemic and inflammatory stages. The details of our mathematical model and computational solution method are discussed in this section.

### Mathematical model and solution method

Heat transfer in human tissue can be modeled using the well-established bioheat equation, introduced by Pennes in 1948 [23]. It is a transient heat conduction equation of the form

(1) $$\mathrm{\rho c}\frac{\partial T}{\partial t}=\nabla \cdot \left(k\nabla T\right)+\omega_{b}\rho_{b}c_{b}\left(T_{b}-T\right)+q,$$

where *ρ, c*, *k*, *q, T* are the local tissue density (kg/m^3^), specific heat (J/kg∙K), thermal conductivity (W/m∙K), metabolic heat generation rate (W/m^3^) and tissue temperature (°C) respectively. *ω*_*b*_, *ρ*_*b*_*, c*_*b*_ and *T*_*b*_ represent the rate of tissue blood perfusion (1/s), blood density (kg/m^3^), blood specific heat (J/kg∙K) and arterial blood temperature (°C), respectively. The equation describes the rate of change of thermal energy within a differential control volume of the tissue as being equal to the rate of the net amount of heat that leaves/enters the control volume by heat conduction, blood perfusion and metabolic heat generation.

We modeled the heel tissue cross section in the form of a two-dimensional (2D) semi elliptical domain, as shown in Figure 3b. The heel consists of six tissue layers: epidermis (1), papillary dermis (2), reticular dermis (3), fat (4), muscle (5) and bone (6). The elliptical lesion represents the DTI (7) in Figure 3b (note that this schematic is not to scale and the thicknesses of the tissue layers are overemphasized to clearly show the anatomical details). Each layer is assumed to be homogenous and to have a uniform thickness. Metabolic heat generation rate, blood perfusion rate and thermal conductivity in each tissue layer were also assumed to be uniform. A set of seven coupled differential equations (one equation for each domain) of the form

(2) $$\begin{array}{l} \rho_{i}c_{i}\frac{\partial T_{i}}{\partial t}=\nabla \cdot \left(k_{i}\nabla T_{i}\right)+\omega_{b_{i}}\rho_{b_{i}}c_{b_{i}}\left(T_{b_{i}}-T_{i}\right)+q_{i}\\ \;i=1,2,3,4,5,6,7, \end{array}$$

together with appropriate initial, boundary and interface conditions describe the mathematical model of this problem. Skin layer thicknesses, lesion dimensions and thermophysical properties used in the models are summarized in Tables 1 and 2. Nominal values of skin tissue thermophysical properties were selected based on previously published data [31,32].

**Table 1:** **Thermophysical properties of heel tissue used in the simulations (all data is taken from**[24]**unless otherwise stated)**

	***Thickness (mm)***	***Specific heat (J/kg·K)***	***Thermal conductivity (W/m·K)***	***Perfusion rate (10***^***-3***^ ***) (1/s)***	***Metabolic heat generation (W/m***^***3***^ ***)***	***Arterial blood temperature (°C)***	***Density (kg/m***^***3***^ ***)***
Epidermis	0.46 [25]	3589	0.235	0	0	-	1200
Papillary dermis	1.67 [25]	3300	0.445	0.18	368.1	37	1200
Reticular dermis	1.67 [25]	3300	0.445	1.26	368.1	37	1200
Fat layer	5 [25]	2674	0.185	0.08	368.3	37	1000
Muscle	2.5 [25]	3600	0.51	2.7	684.2	37	1085
Bone	-	1300 [26]	0.4 [26]	0 [27]	0 [27]	-	2000 [26]

**Table 2:** Thermophysical properties of DTI used in the simulations

***DTI***	***Size of lesion (cm)***	***Specific heat (J/kg·K)***	***Thermal conductivity (W/m·K)***	***Perfusion rate (10***^***-3***^ ***) (1/s)***	***Metabolic heat generation (W/m***^***3***^ ***)***	***Arterial blood temperature (°C)***	***Density (kg/m***^***3***^ ***)***
Ischemia	d_1_= 1.5 [28] d_2_=0.25 [28]	2450 [29]	0.1 [29]	0.405 [15]	342.1 [29]	35 [30]	1037 [29]
Inflammation	d_1_= 1.5 [28] d_2_=0.25 [28]	2450 [29]	0.558 [24]	6.95 [24]	5262.5 [24]	37 [24]	1037 [29]

**Figure 4:**
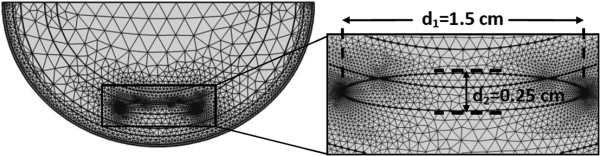
**Computational mesh for the heel cross section aa’ from Figure**3**with an 8 mm deep elliptical DTI lesion with d1 and d2 being the major and the minor axis, respectively.** The mesh consists of 4932 free triangular elements.

The system of seven equations, Equations 2 is solved using the conditions of the continuity of temperature

(3) $$\begin{array}{l} {\left(T_{m}\left(x,y,t\right)\right|}_{j_{m}}={\left(T_{m+1}\left(x,y,t\right)\right|}_{j_{m}},\\ \;m=1,2,3,4,5,6, \end{array}$$

and heat flux

(4) $$\begin{array}{l} {\left(-k_{m}\frac{\partial T_{m}\left(x,y,t\right)}{\partial n}\right|}_{j_{m}}={\left(-k_{m+1}\frac{\partial T_{m+1}\left(x,y,t\right)}{\partial n}\right|}_{j_{m}},\\ \;m=1,2,3,4,5,6, \end{array}$$

at the interfaces between tissue layers, where n is the direction of the normal to a boundary. The boundary condition of zero heat flux is used at the top horizontal surface of the domain (Figure 3b). It can be seen from Figure 4 that the distance between the lesion center and the top surface is sufficiently large (2.2 cm) for the top surface to feel the thermal influence of the lesion. Adding to this, the heat flux from the skin layers is in the radial direction and a uniform core body temperature is assumed at the muscle-bone interface. The boundary condition for the top horizontal surface is given by

(5) $${\left(-k\frac{\partial T\left(x,y,t\right)}{\partial n}\right|}_{\mathrm{top}\;\mathrm{surface}}=0.$$

The interface between the muscle and the bone was assumed to be at constant core body temperature

(6) $${\left(T\left(x,y,t\right)\right|}_{j_{5}}=37℃.$$

Thermal modeling is performed in three steps: (i) computation of the steady state temperature distribution with the skin surface exposed to ambient conditions, (ii) application of a cooling load on the skin surface, and (iii) computation of thermal recovery temperature distributions of the skin surface as a function of time after the removal of cooling (the skin surface exposed to ambient conditions again). The thermal boundary condition at the skin surface for the steady state situation with skin exposed to ambient air is

(7) $$\begin{array}{c} q"=h_{\infty }\left(T\left(x,y,t\right)|{}_{\mathrm{skin}}-T_{\infty }\right),\;t<0\;\mathrm{and}\;t>t_{c},\\ \mathrm{with}\;h_{\infty }=12\;\frac{W}{m^{2}\;K}\;\mathrm{and}\;T_{\infty }=22℃. \end{array}\;$$

The temperature distribution determined for the steady state solution was used as the initial condition for the transient cooling process. The steady state temperature distribution in the tissue not only predicts its thermal state before the cooling begins, but also describes the state that it gradually attains during thermal recovery process [31]. In the transient analysis we applied cooling to the skin surface to examine the thermal recovery of both healthy tissue and tissue with DTI. At time *t* = 0 the constant temperature boundary condition

(8) $${\left(T\left(x,y,t\right)\right|}_{\mathrm{skin}}=15{}^{\circ}C,\;0\leq t<t_{c},$$

was applied on the skin surface to achieve cooling. In this study the cooling duration *t*_*c*_ = 1 min was selected. This boundary condition can be approximated in a clinical setting by applying a cold gel pack on the skin surface for the duration of cooling that is optimized to minimize patient discomfort and scan time. After the cooling was removed at time *t* = 1 min, the convective boundary condition described by equation (7) was applied again and the skin surface was allowed to recover its steady state temperature. The temperature distribution found at the end of the cooling process was used as the initial condition for the transient thermal recovery process. The complete thermal recovery takes around 25–30 minutes, however the largest temperature differences are detected during the first few minutes. Therefore this initial thermal recovery time interval is the one of interest for clinical diagnostic applications.

Computations were carried out using the finite element software COMSOL Multiphysics v4.3a. The mesh consisted of 4932 free triangular elements. Figure 4 shows the computational mesh along with the magnified region of the lesion and its surroundings. Temperatures at the skin surface and around the lesion were of primary interest in the study. To achieve high spatial resolution, the maximum element size for the epidermis, the muscle and fat layers was set as 1.3 mm, whereas the maximum mesh size was 2.4 mm for the rest of the domains. The computational results differed less than 1% when the smallest element size was set as 0.65 mm. Convergence of the solution was ensured by setting the time step ∆t as 0.1 s for the first 5 minutes of thermal recovery and as 1 s for the rest of the thermal recovery period.

### Thermal staging of DTI

In this study, the early stages of a DTI lesion are characterized either by ischemia or inflammation, and thermal models were built for these two cases. Table 2 summarizes the thermophysical properties for these two DTI models. A multilayer model was also built to account for later stages of DTI. Staging the injury in terms of ischemia and inflammation is useful because it helps to explain the inconsistency in previously reported thermographic measurements of pressure ulcers. Temperatures computed for these models were compared with temperatures from the healthy tissue model. In order to evaluate the impact of lesion depth on surface temperature distributions, we considered three cases of subcutaneous tissue damage in heel DTI: an 8 mm deep lesion representing damage in the muscle and fat layers (Figure 3c), a 6 mm deep lesion representing damage in fat layer alone (Figure 3d) and a 3.8 mm deep lesion representing damage restricted to reticular dermis (Figure 3e).

### Healthy tissue model

This model serves as the baseline model for comparisons between healthy tissue and tissue with DTI. The healthy heel model does not have any lesion and has normal metabolic functioning. The thermophysical properties for healthy tissue used in the mathematical model are summarized in Table 1.

### Ischemia model

This model predicts temperature distributions in the heel tissue in the presence of deep tissue ischemia. In a pressure related ischemic injury, partial or complete occlusion of the blood vessels occurs when the magnitude of external pressure exceeds the capillary closing pressure of 32 mm Hg [14], resulting in decreased perfusion levels [15,33]. Decrease in metabolic heat generation rate is postulated to occur as the tissue does not get sufficient oxygen to sustain aerobic metabolic processes (also shown in Figure 1d). Injury to the deep tissue causes lipid accumulation in the degenerated tissue resulting in lower density [28,29] and lower thermal conductivity [29] values as compared to the healthy tissue. The thermophysical properties of the lesion during ischemia are summarized in Table 2.

### Inflammation model

This model predicts temperature distributions in the heel tissue in the presence of inflammatory processes which occur after an ischemic injury. Laser doppler flowmetry was used in a rat model to measure changes in skin perfusion levels in response to incremental pressure loading [15]. An initial rise in blood perfusion was observed during tissue loading phase (when pressure levels were within tolerable limits). A larger second peak was caused by ischemic reperfusion of the tissue and occurred after the pressure loading was removed. Inflammation may occur early in the form of repair mechanisms which are initiated by the adjacent healthy tissue (also shown in Figure 1e or during post ischemic reperfusion when inflammatory mediators are generated by metabolic processes [19] (also shown in Figure 2d). In this model, we characterized the lesion as a region of increased blood perfusion and increased metabolic heat generation. Table 2 summarizes the thermophysical properties of the lesion during inflammation, and the data indicate significant increase in thermal conductivity, perfusion rate and metabolic heat generation.

### Multilayer DTI model

We define the multilayer DTI as a lesion which consists of an inner ischemic region (core) surrounded by an outer inflammation layer. Our multilayer DTI models are representative of an injury phase during which the accumulation of injured tissue (which may have been caused by an ischemic insult) initiates an inflammatory response in the surrounding tissue (also shown in Figure 1k) and it arises at later stages of the DTI.

## Results

Tissue with DTI can be distinguished from healthy tissue by cooling down the skin surface and measuring the thermal recovery as a function of time, as introduced in the mathematical model. While the steady state situation yields relatively large temperature differences that are easy to measure, even more information can be gained and the temperature difference can be enhanced (when needed, for example for small lesions) by considering transient thermal recovery temperatures. In the present study the skin surface was cooled down to 15°C, and its transient thermal recovery was computed using the two DTI models.

**Figure 5:**
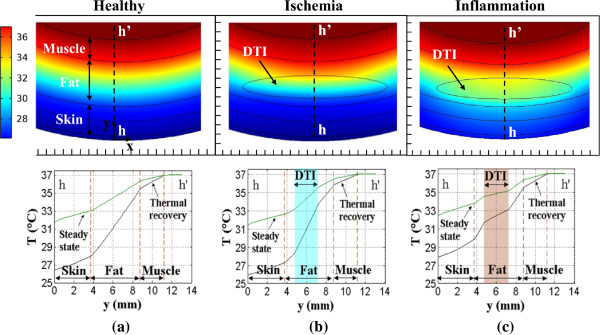
**Color coded 2D temperature distributions (top row) and temperature profiles plotted along hh’ (bottom row) for (a) healthy tissue and (b) tissue with a 6 mm deep DTI for DTI ischemia and (c) inflammation.** Temperature distributions are computed at 3.5 minutes into the thermal recovery, at a time when the temperature differences of interest are large.

Figure 5 shows color-coded 2D heel tissue temperature distributions (top row of images) computed by solving healthy tissue and DTI models for ischemia and inflammation (Eqs. 2, 3, 4, 5, 6, 7 and 8) at 3.5 minutes into the thermal recovery process. This time is selected because the temperature difference between healthy tissue and lesion is largest in the 3-4 min time range. The blue regions in the color-coded images correspond to lowest temperature and red regions to highest temperature. Figure 5a shows 2D temperature distributions and temperature profiles in the healthy heel tissue, whereas Figure 5b and 5c illustrate the tissue temperature variations for DTI ischemia and DTI inflammation, respectively. In the color coded temperature images a temperature increase is visible in the region of the lesion and its surroundings for inflammation, whereas the temperature decreases in this region for ischemia, when compared to healthy tissue.

Tissue temperature is plotted as a function of tissue depth along the line hh’ in the bottom row of images in Figure 5. The black plots show the transient temperatures at 3.5 minutes of thermal recovery and the green plots show the steady state temperatures. The skin surface is at h = 0 mm and h’ = 11.3 mm is the location of the bone-muscle interface. For a 6 mm deep lesion, while the steady state situation yields relatively large temperature differences at the skin surface (h = 0 mm) that are easy to measure (temperature decrease of 0.35°C during ischemia and increase of 0.6°C during inflammation), the temperature differences can be enhanced by considering transient temperatures during thermal recovery. During transient recovery (at 3.5 minutes), a temperature decrease of 0.41°C is observed during ischemia and a temperature increase of 0.7°C is observed during inflammation. Both the 0.41°C temperature decrease and the 0.7°C temperature increase (with respect to the healthy situation) at the skin surface (h = 0 mm) can easily be measured with modern, relatively low cost IR cameras. The multilayer DTI model results (which will be discussed later) show that even more information can be obtained by considering thermal transient temperatures.

**Figure 6:**
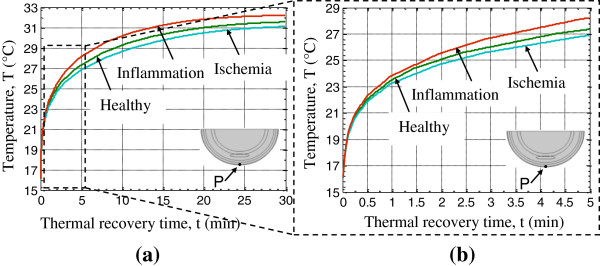
**Temperature of point P on the skin surface (P shown in the schematic) during thermal recovery for healthy tissue, tissue with ischemia and tissue with inflammation. (a)** Complete thermal recovery – until temperature of the skin reaches steady state and **(b)** magnified region of the early thermal recovery period – the first five minutes of particular interest in clinical applications.

These two transient thermal responses were compared with those of healthy tissue, as illustrated in Figure 6. The point P at which the temperatures were computed (located on the skin surface below the center of the DTI lesion) is shown in the geometry schematic in Figure 6. The results suggest that the skin surface temperature is lower than the healthy skin in the presence of deep lesion ischemia and higher than the healthy skin during inflammation. Complete thermal recovery takes about 20–25 minutes (Figure 6a) for a cooling load of 15°C of 1 minute duration, however, key information regarding the nature of the DTI is available within the first 2–4 minutes of thermal recovery (Figure 6b). Therefore the duration of a transient measurement would be 1-5 minutes, depending on the location and size of the lesion as well as tissue properties, which is acceptable in a clinical setting. The thermal recovery of the skin surface for the first five minutes, of interest in a clinical application, is shown in the magnified region in Figure 6b. For an 8 mm deep lesion, the temperature difference for ischemia ∆T_isc_ (T_ischemia_ – T_healthy_) is in the range −0.25°C - −0.5°C. The temperature difference for inflammation ∆T_inf_ (T_inflammation_-T_healthy_) is 0.5°C - 0.9°C during the first five minutes. Current modern IR cameras and our dynamic thermal imaging system [34] can be used to measure such temperature differences to identify thermal changes associated with deep tissue ischemia and inflammation.

**Figure 7:**
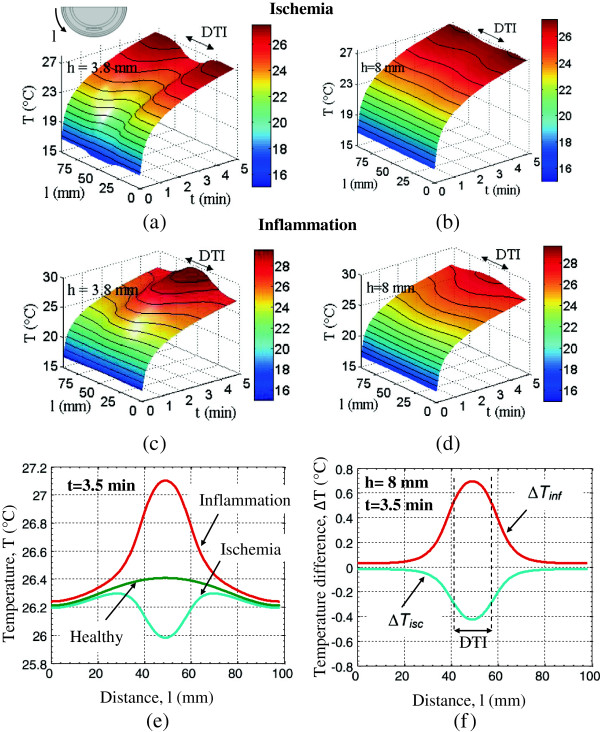
**Skin surface temperature distribution as function of location and time for DTI lesions.** Ischemia lesions at **(a)** h=3.8 mm and **(b)** h=8 mm depth and inflammation lesions at **(c)** h=3.8 mm and **(d)** h=8 mm depth. **(e)** Temperature profiles along the skin surface for healthy tissue and tissue with an 8 mm deep DTI and **(f)** temperature differences ∆T_inf_ = T_inflammation_-T_healthy_ and ∆T_isc_ = T_ischemia_-T_healthy_ along skin surface at t = 3.5 min into the thermal recovery.

Figure 7 shows the spatial dependence of the skin surface temperatures along the heel periphery in the presence of lesion ischemia and inflammation. The distance l, measured along the heel periphery, is indicated in the schematic in Figure 7a (top). The skin temperature is plotted as a function of the thermal recovery time for two lesion depths, h=3.8 mm (Figures 7a and 7c) and h=8 mm (Figures 7b and 7d), as illustrated in color coded 3D temperature plots in Figures 7a-d. The location where the maximum temperature decrease or increase is observed with respect to the healthy tissue at a given recovery time corresponds to the distance between 35–55 mm along the heel periphery (Figure 7e), which is the area immediately underneath the lesion. For an 8 mm deep ischemia lesion, at 3.5 minutes into thermal recovery, the maximum temperature decrease between healthy tissue temperature and the ischemic region was observed to be 0.41°C. The maximum temperature increase for inflammation was 0.7°C (Figure 7f). Again, these temperature differences can be measured using modern IR cameras.

**Figure 8:**
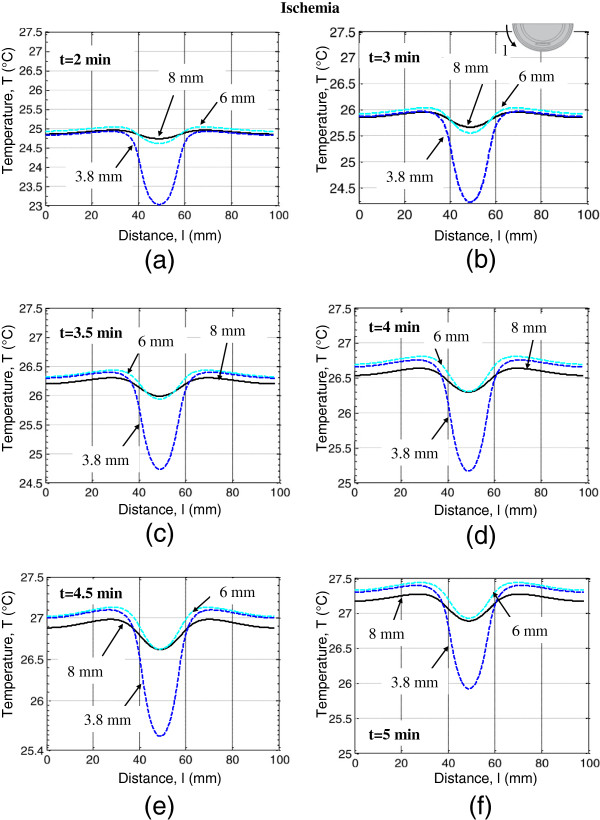
Skin surface temperature profiles plotted along the heel periphery for DTI ischemia lesions at depths h=3.8 mm, 6 mm and 8 mm for thermal recovery times (a) t=2 min, (b) t=3 min, (c) t=3.5 min, (d) t=4 min, (e) t=4.5 min and (f) t=5 min.

**Figure 9:**
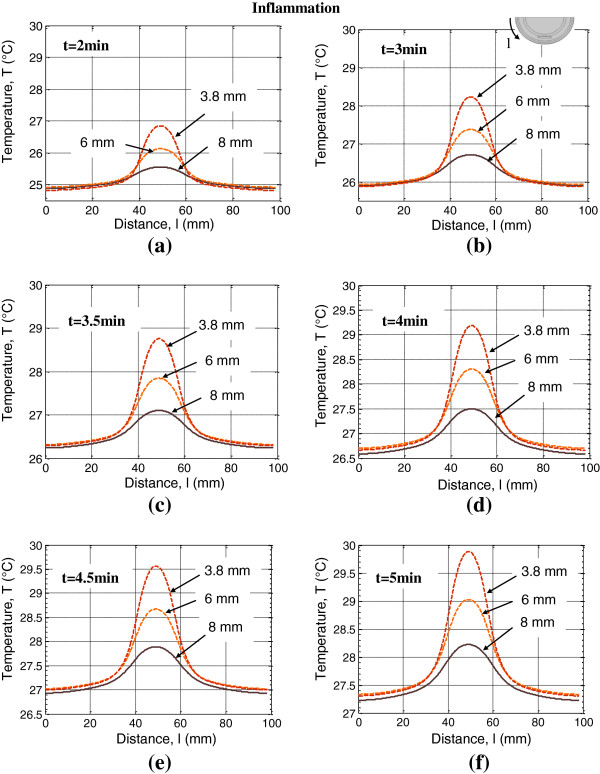
Skin surface temperature profiles plotted along the heel periphery for DTI inflammation lesions at depths h=3.8 mm, 6 mm and 8 mm for thermal recovery times (a) t=2 min, (b) t=3 min, (c) t=3.5 min, (d) t=4 min, (e) t=4.5 min and (f) t=5 min.

To analyze the impact of lesion depth on the surface temperature signatures, we modeled DTI ischemia and DTI inflammation for three lesion depths: 3.8 mm (lesion in the fat layer and reticular dermis), 6 mm (lesion in the fat layer) and 8 mm (lesion in the muscle and fat layers). The skin surface thermal response for first few minutes (2–5 minutes) of the thermal recovery process was computed for these lesion depths in the presence of DTI ischemia (Figure 8) and DTI inflammation (Figure 9). The skin surface temperature decrease due to the presence of the ischemic lesion is most pronounced for the lesion closest to the surface, the 3.8 mm deep lesion. For 3.8 mm deep ischemia lesion, the magnitude of temperature decrease at later recovery times is, however, less as compared to its magnitude at t=2 minutes. For lesions at depths of 6 mm and 8 mm, a larger temperature decrease is observed during the first 2–4.5 minutes of thermal recovery, when compared to the decrease at t=5 minutes. The skin surface temperature increases with decreasing depth of the inflammation lesion: as expected, the lesion closest to the skin surface, a 3.8 mm deep lesion results in a higher temperature increase than a 6 mm deep or an 8 mm deep lesion. These results suggest that imaging times of 2–4.5 minutes will be sufficient for best sensitivity in dynamic thermal imaging.

**Figure 10:**
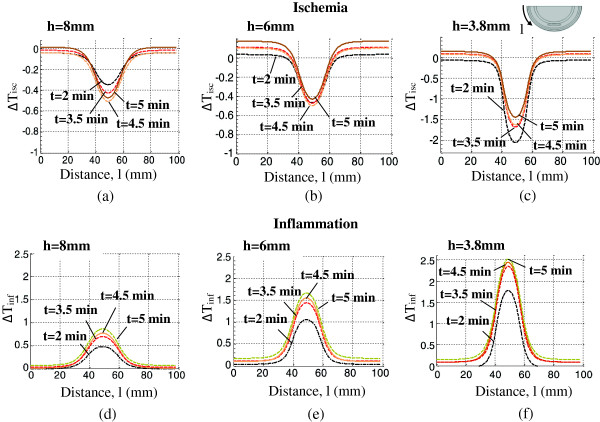
**Temperature difference, ∆T (T**_**DTI **_**–T**_**healthy**_**), on the skin surface observed for t=2, 3.5, 4.5 and 5 minutes into the thermal recovery period for DTI ischemia lesions at (a) h=8 mm, (b) h=6 mm and (c) h=3.8 mm depth as well as for DTI inflammation lesions at (d) h=8 mm, (e) h=6 mm and (f) h=3.8 mm depth.**

Surface temperatures computed using the ischemia and inflammation models were compared with the healthy tissue data, and the temperature difference results are presented in Figure 10. The temperature differences for three depths of DTI ischemia and DTI inflammation lesions are shown for the 2-5 minutes period of thermal recovery. The top row of Figure 10 shows ∆T_isc_ for ischemia and the bottom row shows ∆T_inf_ for inflammation. ∆T_isc_ for an 8 mm deep ischemia lesion, first decreases from −0.38°C to −0.5°C between 2 to 4.5 minutes, and then reaches the value of −0.45°C at 5 minutes. ∆T_isc_ for a 6 mm deep lesion, decreases similarly from −0.45°C to −0.5°C between 2 to 4.5 minutes and then reaches −0.41°C at 5 minutes. For a 3.8 mm deep lesion the minimum of ∆T_isc_ was observed at 2 minutes and was approximately −2°C. It increased from −2°C to −1.4°C at the end of 5 minutes. These results suggest that early times in the thermal recovery process are important in the identification of ischemic DTI lesions.

∆T_inf_ lies in the range of 1.7°C - 2.5°C for a 3.8 mm deep lesion, 1.0°C - 1.6° for a 6 mm deep lesion and 0.25°C - 0.9°C for an 8 mm deep lesion for 2–5 minutes of thermal recovery during inflammation. These data suggest that the thermal signature of the inflammation lesion becomes more pronounced (corresponding to a larger measurement signal) when the lesion is closer to the skin surface, as expected. Early times in the thermal recovery process are also important in the identification of inflammation DTI lesions.

**Figure 11:**
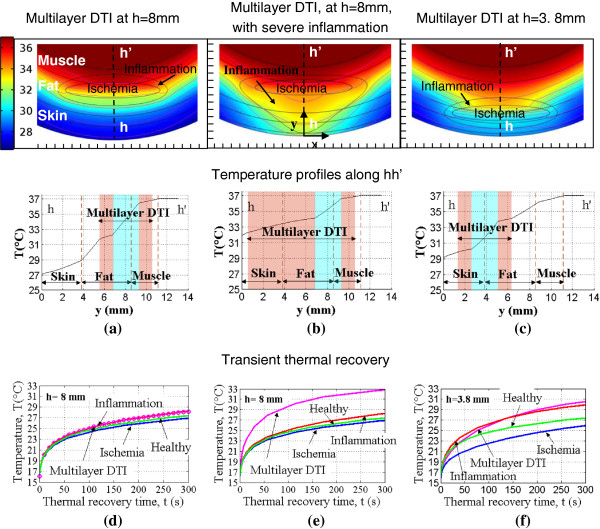
**Temperature distribution in multilayer DTIs.** Color coded 2D temperature distributions (top row) and temperature profiles plotted along the line hh’ (bottom row) for multilayer DTIs for an **(a)** h=8 mm deep lesion with mild inflammation, **(b)** h=8 mm deep lesion with severe inflammation and an **(c)** h=3.8 mm deep lesion with mild inflammation. Temperature distributions are computed at 3.5 minutes into the thermal recovery, at a time when the temperature differences of interest are large. Temperature as a function of time during thermal recovery for healthy tissue, multilayer DTIs, ischemia and inflammation for **(d)** h=8 mm depth and mild inflammation (case (a)), **(e)** h=8 mm depth, severe inflammation (case (b)) and **(f)** h=3.8 mm depth, mild inflammation (case (c)).

Figure 11 shows temperature distributions computed at 3.5 minutes into the thermal recovery for the multilayer DTI models that have an inner ischemic region surrounded by an outer inflammation layer. These models are representative of an injury phase during which the accumulation of injured tissue (which may have been caused by an ischemic insult) initiates an inflammatory response in the surrounding tissue. This situation arises at later stages of the DTI. We modeled three multilayer cases: an 8 mm deep ischemic lesion surrounded by a 1.25 mm thick inflammation layer (Figure 11a); an 8 mm deep ischemic lesion surrounded by severe inflammation which extends up to the skin surface (Figure 11b) – a precursor of a pressure ulcer; and a 3.8 mm deep ischemic lesion that is surrounded by 1.25 mm thick inflammation layer (Figure 11c). Tissue temperature profiles plotted along line hh’ indicate that these lesions cause an increase in the skin surface temperature. Healthy tissue temperature distributions computed at the same recovery time (3.5 min) are shown in Figure 5a and serve as baseline data for the multilayer DTI analysis.

The thermal recovery as a function of time was computed for the three multilayer DTI models and results were compared with healthy tissue as well as DTI ischemia, inflammation results for the same lesion depth (Figures 11d-f). Data in Figures 11d and 11e show that the skin surface temperature is higher during thermal recovery in the presence of 8 mm deep multilayer DTI lesions than for the healthy tissue and the corresponding inflammation and ischemia cases. The difference in temperature during the first five minutes of the thermal recovery period, defined as ∆T (T_multilayerDTI_ – T_healthy_), varied in the range 0.2°C - 0.8°C for the multilayer lesion with 1.25 mm thick inflammation zone (Figure 11d) and between 0.5°C - 5.5°C for the multilayer lesion with severe inflammation (Figure 11e).

**Figure 12:**
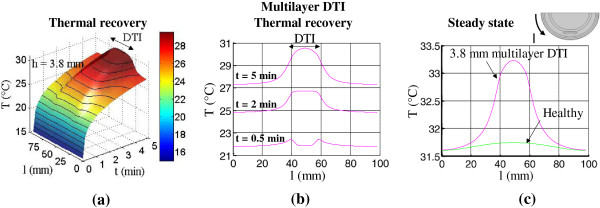
**Skin surface temperature profiles during thermal recovery for an h=3.8 mm deep multilayer DTI. (a)** Temperature profiles for the multilayer DTI shown as a function of location and time **(b)** Temperature profiles at t=0.5, 2 and 5 min during the thermal recovery period. At t=0.5 min the competing effects of ischemia (accompanied by temperature decrease in the central region) and inflammation (accompanied by temperature increase with maxima at the edges of the low-temperature central region) are observed. **(c)** Temperature distribution along the skin surface in steady state for the multilayer DTI and healthy tissue.

In the presence of the 3.8 mm deep multilayer DTI lesion, the skin surface showed both a temperature increase (inflammation dominated thermal signature) and a temperature decrease (ischemia dominated thermal signature) compared to the healthy tissue during the thermal recovery period (Figures 11f and 12a). The surface temperature distribution is a result of the competing effects of temperature decrease, an indication of ischemia, and temperature increase, an indication of inflammation. Temperature decrease is detectable on the skin surface only during the early stages of the transient analysis and not during steady state. The period of temperature decrease was observed during the early thermal recovery period, i.e. for t < 2 minutes (Figure 12b). The maximum temperature decrease was −0.6°C. After this period, temperature increase was observed as the skin surface temperature recovered further (Figure 12b). The maximum increase of 3.2°C was observed within the first five minutes. This is over twice the value of the steady state temperature difference of 1.5°C (Figure 12c) discussed earlier. These results clearly illustrate the possibilities of generating larger temperature differences (measurement signals) and reducing measurement errors by using dynamic IR imaging contrasted to steady state techniques.

## Discussion

A thermographic scan of the tissue at risk for DTI will either show normal skin temperature, a temperature drop or temperature rise, when compared to the temperature of surrounding healthy skin or the healthy tissue temperature of the same body part measured at an earlier time. If at risk patients are monitored over a longer period of time, changes relative to earlier scans can also be considered for the assessment of the potential for DTI and the quantitative assessment of the level of damage caused by DTI. Our computational analysis allows better insight into the processes underlying the temperature changes manifested on the skin surface and better qualitative and quantitative interpretation of data. Our computational thermal modeling results show that tissue ischemia and inflammation can be manifested as skin surface temperature decrease and increase, respectively. Coupling inverse reconstruction techniques to surface temperature data obtained by IR imaging [34,35], key parameters of the DTI lesion, such as dimensions, depth, metabolic heat generation and blood perfusion rates can be estimated [36], which is the subject of our current research efforts. The long term goal of our study is to relate thermographic scan information with key physiological changes in the tissue to allow clinicians to detect DTI in early stages and to provide necessary intervention to mitigate its progression.

**Figure 13:**
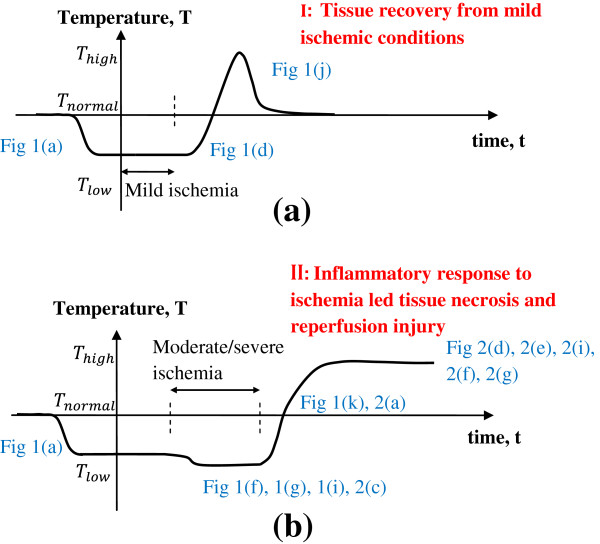
**Qualitative display of skin surface temperature evolution over time for two clinical outcomes of DTI. (a)** Case I: tissue recovers from a brief ischemic episode and **(b)** Case II: tissue suffers irreversible tissue damage as a result of severe inflammatory response and ischemia-reperfusion injury.

We summarize the conclusions from our computational analysis in Figure 13. The plots qualitatively describe the expected long-term skin surface temperature variations during DTI occurrence and progression. Figure 13a shows the evolution of changes of skin surface temperature from a time when the tissue experiences sustained loading to the time the tissue recovers and returns to a healthy state. The initial drop in skin surface temperature will be the result of a mild DTI ischemia lesion. Case I in Figure 13a accounts for the possibility of tissue recovery after a brief ischemic episode. The thermoregulation processes (Figure 1e), will be able to restore normal perfusion levels and recover the tissue to its healthy state. The magnitude of temperature drop on the skin surface during this period will depend on the extent of ischemic damage, lesion dimensions and depth.

The possibility of severe inflammatory response (resulting from ischemic changes which lead to tissue necrosis) and ischemia reperfusion mediated injury are considered in Case II (Figure 13b). The plot in Figure 13b shows the evolution of skin surface temperature during the course of irreversible tissue damage. Moderate to severe ischemic conditions (Figures 1f, 1g, 1i, and 2c) will result in a DTI ischemia lesion and a more pronounced temperature decrease. When post ischemic revascularization occurs, the injury may start out as a single thin inflammation layer around the ischemic region and transform into a multilayer DTI (Figure 1k) or into a DTI inflammation lesion (Figure 2a) over time. A sustained temperature increase is possible due to reperfusion cascade (Figure 2d-g and 2h). Further research is necessary to examine the time scales over which temperature decrease and temperature increase would be observed. Furthermore, the impact of larger scale tissue necrosis with substantial cell death seen in later stages of DTI needs to be evaluated in terms of thermographic monitoring.

Early detection of DTI using IR thermography will benefit from regular thermographic monitoring of the patient. Also important is the time when the patient is imaged for the first time: this time may or may not coincide with the onset of deep tissue damage. It is thus recommended to identify high risk patients as early as possible and begin thermographic monitoring of the skin surface temperature early.

## Conclusions

We have demonstrated that a computational heel DTI thermal model can explain previously published thermographic findings related to DTI and pressure ulcers. Ischemia and inflammation have been included as thermal stages of early DTI in our computational model. Staging the injury in this manner helps to explain the seemingly inconsistent data in prior published thermographic measurements of pressure ulcers. We demonstrate that tissue with early DTI, inflammation, ischemia or multilayer DTI, can be identified by measuring either steady state temperature distributions or the thermal response of the skin surface to a cooling stress and comparing it with the thermal response of the healthy tissue. Ischemia is characterized by decrease in skin surface temperature and ischemia-reperfusion injury associated with inflammation is characterized by an increase in skin surface temperature. For a deep lesion situated in muscle and fat layers, a temperature increase between 0.25°C to 0.9°C during inflammation can be expected, contrasted to a temperature decrease between −0.2°C to −0.5°C during ischemia – observed within the first five minutes of thermal recovery period. Given the magnitude of these temperature differences, thermographic imaging can be used to identify early inflammation and ischemic changes associated with impending infarction. The computational model will help the clinicians to relate thermographic findings with key physiological changes, to identify patients with risk at an early stage of the injury, and provide necessary intervention to prevent the spreading of the lesion. A sensitivity analysis of skin surface temperature distribution to parameters such as thermophysical properties, blood perfusion rate, metabolic heat generation rate, and tissue layer thicknesses is the next step to better characterize early DTI lesions.

## Abbreviations

DTI: Deep tissue injury; IR: Infrared; 2D: Two-dimensional; ATP: Adenosine triphosphate; ROS: Reactive oxygen species; NPUAP: National pressure Ulcer advisory panel.

## Competing interests

The authors declare that they have no competing interests.

## Authors’ contributions

AB and CH designed the study, obtained and analyzed the data. CH, AB and AC contributed in interpreting the data and in drafting and reviewing the manuscript. All authors read and approved the final manuscript.

## Authors’ information

AC is an associate professor in the Department of Emergency Medicine at the Johns Hopkins University School of Medicine. He is currently a director of an emergency department and was the residency director for the Emergency Medicine Residency Program at the Johns Hopkins Hospital from 2000 to 2010. He is the editor and associate editor for a number of textbooks in Emergency Medicine and is a regular contributor and medical director for Practical Reviews, a monthly publication.

CH is Professor in the Department of Mechanical engineering at the Johns Hopkins University. Her area of expertise is the application of optical measurements in engineering and biomedical systems. She has been focusing on infrared thermography over the past decade and her research on melanoma diagnostics using dynamic infrared technique has been reported in the science literature.

AB received her M.S. degree from the Department of Mechanical Engineering at the Johns Hopkins University in 2013 and is currently pursuing her PhD. Her research interests include computational modeling in bioheat transfer and infrared thermography.
